# The herpesvirus stealth program

**DOI:** 10.18632/oncotarget.5261

**Published:** 2015-08-26

**Authors:** Teng Huang, Nikolaus Osterrieder

**Affiliations:** Institut für Virologie, Zentrum für Infektionsmedizin - Robert von Ostertag-Haus, Freie Universität Berlin, Berlin, Germany

**Keywords:** Immunology and Microbiology Section, Immune response, Immunity, herpesvirus, MHC I, downregulation, endocytosis, degradation

Immune evasion represents a critical strategy that many viruses have evolved to ensure survival in individual hosts and entire populations. In vertebrates, particularly mammals that are protected by a highly sophisticated immune system, viruses need to antagonize the many defense line drawn after antigen recognition and the ensuing differentiation and proliferation of virus-specific T and B cells.

The *Herpesviridae* family is characterized by complex viral genomes that contain a number of so-called non-essential genes, which are dispensable for growth in cultured cells but not *in vivo*. It is no surprise that herpesviruses encode, among many other immune evasion factors, gene products that target antigen presentation by major histocompatibility complex class I (MHC-I) [1]. Due to the drastic reduction of cell surface MHC-I molecules, the signals that activate and recruit cytotoxic T lymphocytes (CTLs) to the infected cell are blocked. As a result, the infected cells cannot be eliminated and thus become a reservoir for the sustained production and release of viruses.

**Figure 1:**
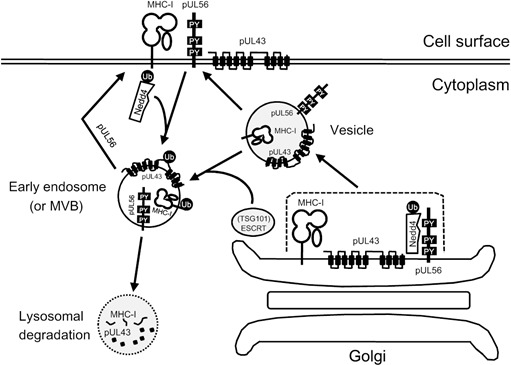
Model of the presumed cooperation between EHV-1 pUL43 and pUL56 in MHC-I downregulation

The viral inhibitors that cause downregulation of MHC-I on the cell surface can vary widely with respect to mechanistic principles, even among closely related virus species. For instance, some pUL49.5 homologues from the *Varicellovirus* genus of the *Alphaherpesviridae* have been shown to interfere with the MHC-I presentation pathway. The viral proteins block the physiological activity of the transporter associated with antigen processing (TAP) that facilitates translocation of peptides produced by the proteasome into the endoplasmic reticulum (ER) and ultimately loading of antigenic peptides onto MHC-I. Regardless of the structural similarities, there are remarkable differences in the molecular functions exerted by pUL49.5 homologues. In the case of bovine herpesvirus 1 (BoHV-1), the TAP complex is tagged for proteasomal degradation by pUL49.5, whereas the pUL49.5 homologues of equine herpesvirus 1 (EHV-1) and EHV-4 impair the function of the TAP complex merely through inhibition of ATP binding (reviewed in [2]). Apart from the pUL49.5 homologues, the EHV-1 pUL56 was shown to be involved in downregulation of cell surface MHC-I [3]. Detailed studies revealed that early in infection the expression of pUL56 correlates with internalization of cell surface MHC-I through dynamin-dependent endocytosis, a process that requires activation of the cellular ubiquitination cascade [4]. Knowing that pUL56 suppresses the MHC-I presentation only during viral infection and not by itself, we speculated that there is at least one viral interactor of pUL56. We, therefore, generated a library of single knockout mutants and attempted to find partner(s) for pUL56. Our investigation resulted in identification of a poorly understood protein equivalent to the HSV-1 (Herpes simplex virus 1) pUL43 homologue that is also involved in the inhibition of the presence of MHC-I molecules on the cell surface. Most importantly, direct interaction of pUL43 and pUL56 was demonstrated, indicating that cell surface MHC-I levels are reduced by cooperation of the two transmembrane proteins [5]. The putative mechanism of MHC-I downregulation induced by the pUL43-pUL56 cooperation is illustrated in Figure 1. Briefly, we presume that ubiquitination of pUL43 is initiated by the interaction between pUL56 and Nedd4 (Neural precursor cell expressed developmentally down-regulated protein 4), which then directs delivery of the internalized tertiary complex MHC-I-pUL43-pUL56 to the early endosome. The cargo eventually traffics to the lysosomal compartments where MHC-I and pUL43 are degraded. We have shown that pUL56 remains stable during infection [3], which indicates that it is excluded from the tertiary complex at the transition from the endosomal to the lysosomal stage. An alternative model would require vesicles derived from the Golgi apparatus to be directed to the early endosome or MVB (Multivesicular body) by the ESCRT machinery (Endosomal sorting complex required for transport) that would then sort MHC-I and pUL43 for lysosomal degradation. During the degradation process, pUL56 late domains (Pro-Pro-x-Tyr motif; x indicates any amino acid) seem critical for bringing the E3 ligase Nedd4 in close proximity with pUL43 and MHC-I. It will be of importance to test if the identified mechanism of early MHC-I degradation is also active in human cells after infection with the EHV-1 cousins HSV and VZV (Varicella-zoster virus). Both viruses encode pUL43 homologues that share structural similarity with that of EHV-1.

The identification of a novel potent herpesviral inhibitory mechanism of MHC-I presentation suggests that our knowledge about the arsenal of herpesviruses with respect to escape from host immunity is likely far from complete. The continued adaptive selection of countermeasures to host immune responses is in need of a diversity of viral inhibitors that effectively silence or avoid effector cell populations such as macrophages, natural killer (NK) cells, T and B cells. Therefore, the cooperative action of two viral proteins in escape from CTL responses summarized here is likely not to be the last to be identified.
